# Neonatal diabetes as an isolated manifestation of ipex: an expanding spectrum of disease phenotype with FOXP3 mutation

**DOI:** 10.1186/1687-9856-2015-S1-P11

**Published:** 2015-04-28

**Authors:** Mary Abraham, Richard Loh, Catherine Cole, Sharron Townshend, Jayne Houghton, Andrew Hattersley, Elizabeth Davis

**Affiliations:** 1 Department of Endocrinology, Princess Margaret Hospital, Perth, WA, Australia; 2 Department of Immunology, Princess Margaret Hospital, Perth, WA, Australia; 3 Department of Hematology and Oncology, Princess Margaret Hospital, Perth, WA, Australia; 4 School of Paediatrics and Child Health, UWA, Perth, WA, Australia; 5 PathWest Haematology, Perth, WA, Australia; 6 Genetic Services of Western Australia, Princess Margaret Hospital & King Edward Memorial Hospital, WA, Australia; 7 Institute of Biomedical and Clinical Science, University of Exeter Medical School, Exeter, UK

## 

Neonatal diabetes occurs due to a genetic form of pancreatic βcell dysfunction. Mutations in a number of genes have been identified in the last decade which has helped not only in the etiological diagnosis but has also influenced medical therapy.

Our proband was born at term with a birth weight of 3.99kg with no antenatal risk factors. He presented in severe diabetic ketoacidosis at 8 weeks which required intensive management. He was commenced on continuous subcutaneous insulin infusion for his ongoing management. His initial genetic analysis for ABCC8, KCNJ11, INS gene mutations was negative.

He demonstrated excellent glycaemic control with an average HbA1C of 6.5% at 2 years of age. His growth and development are appropriate for age with no associated co-morbidities. However, further targeted next generation sequencing of all known neonatal diabetes genes identified a hemizygous FOXP3 missense mutation p.R347H with his mother as a carrier. FOXP3 mutations cause IPEX syndrome, (immunodysregulation, polyendocrinopathy, enteropathy, X-linked syndrome) and can be lethal in infancy and early childhood. Prophylactic bone marrow treatment is the only known cure for this condition.

Our proband has isolated neonatal diabetes with no other features of IPEX. His maternal grandfather aged 63 years, was also hemizygous for the mutation. He had enteropathy secondary to ulcerative colitis since childhood which was resistant to conventional treatment and required colostomy. This makes him the oldest living man with IPEX. Our proband’s maternal cousin also had eczema in infancy which has resolved.

This case highlights the impact of genetic diagnosis on the management of neonatal diabetes. A lethal condition with no cure other than bone marrow transplant has now been shown to have atypical manifestations with the availability of genetic testing. The management in our patient is challenging given the poor genotype – phenotype correlation and the dearth of information regarding milder forms limited to a few case reports with similar mutation. We have decided against prophylactic bone marrow transplant and will consider it in the event of enteropathy or another autoimmune manifestation.

Written informed Consent for this patient has been taken including results of the genetic analyses according to the Institutional Ethics Committee procedures of our health service

